# Dairy veterinarians’ skills in motivational interviewing are linked to client verbal behavior

**DOI:** 10.1017/S175173112000107X

**Published:** 2020-10

**Authors:** C. Svensson, L. Forsberg, U. Emanuelson, K. K. Reyher, A. M. Bard, S. Betnér, C. von Brömssen, H. Wickström

**Affiliations:** 1 Department of Clinical Sciences, Swedish University of Agricultural Sciences, P.O. Box 7054, SE-750 07 Uppsala, Sweden; 2 MIC Lab AB, Drottninggatan 55, SE-111 21 Stockholm, Sweden; 3 The Bristol Veterinary School, University of Bristol, Langford House, Langford, North Somerset BS40 5DU, UK; 4 Department of Energy and Technology, Unit of Applied Statistics and Mathematics, Swedish University of Agricultural Sciences, P.O. Box 7032, SE-750 07 Uppsala, Sweden; 5 MeetMe Psykologkonsult AB, Åvägen 16, SE-443 31 Lerum, Sweden

**Keywords:** veterinarian–client communication, Change Talk, herd health management, cattle, Client Language Easy Rating

## Abstract

Veterinarians often give advice in a persuasive form, a style that has been shown to evoke resistance to change in clients experiencing psychological ambivalence (i.e. those who see both advantages and disadvantages to changing). With this style of communication, veterinarians run the risk of counteracting their purpose to encourage clients to follow recommendations. Motivational interviewing **(MI)** is a client-centered communication methodology that aims to facilitate clients’ internal motivation to change. In MI, *Change Talk* represents clients’ own statements expressing consideration of, motivation for or commitment to behavior change and has been shown to be strongly correlated with behavior change. *Sustain Talk* is corresponding statements related to maintaining the *status quo*. The aim of this exploratory study was to evaluate the potential of MI to facilitate behavior change in veterinary herd health management **(VHHM)** by investigating the effect of dairy cattle veterinarians’ MI skills on client *Change* and *Sustain Talk*. We recorded VHHM consultancies on 170 Swedish cattle farms performed by 36 veterinarians, randomly distributed into 2 groups: MI veterinarians (*n* = 18) had received 6-month training in MI and control veterinarians (*n* = 18) had not received any training. Veterinarians’ MI skills were assessed using the Motivational Interviewing Treatment Integrity coding system 4.2.1 and categorized as poor_untrained, poor_trained, near moderate and moderate. Client communication was coded using the Client Language Easy Rating coding system. The effect of MI skills on *Change Talk, Sustain Talk* and *Proportion of Change Talk* *(Change Talk* divided by the sum of *Sustain Talk* plus *Change Talk)* was investigated using cross-classified regression models with random intercepts for veterinarian and client (farm). The models also included additional explanatory variables (e.g. type of veterinarian and client’s satisfaction with the consultation). The veterinarian’s MI skills were associated with the client’s *Change Talk,* but results regarding *Sustain Talk* or *Proportion of Change Talk* were inconclusive. Clients of veterinarians reaching the highest (i.e. moderate) MI skills expressed 1.5 times more *Change Talk* than clients of untrained veterinarians. Clients of general large animal practitioners expressed less *Sustain Talk* than clients of animal health veterinarians and had higher *Proportion of Change Talk.* Results indicate that learning to practice MI may be one means to improve adherence to veterinary recommendations and to improve efficiency in VHHM services.

## Implications

We investigated communication between clients and veterinarians with different skills in the client-centered communication methodology motivational interviewing. Clients of veterinarians with the highest skills in motivational interviewing (from among the sampled veterinarians) spoke most favorably about behavior change. Such communication (in favor of change) previously has been shown to be correlated with clients later changing behavior. This finding therefore indicates that learning motivational interviewing may be a means by which veterinarians can inspire farms to implement preventive measures to improve animal health. Herd health advisory services should be revised so that veterinarians give further attention to client motivation and perspectives.

## Introduction

Communication skills are increasingly being acknowledged as important in the veterinary profession (Cake *et al.*, 2016; Ritter *et al.*, 2019). Veterinary herd health management **(VHHM)** services constitute an increasing proportion of work for cattle veterinarians and often focus on changing management routines (i.e. behavior change). In these types of services, change-orientated communication skills therefore may be of special importance. A shared power elicited by relationship-oriented communication and use of a high proportion of empathy statements has been demonstrated to be positively associated with behavior change (Kanji *et al.,* 2012; Moyers and Miller, 2013). However, veterinarians working in VHHM have been found to show very few of these behaviors (Bard *et al.,* 2017; Ritter *et al.,* 2018; Svensson *et al.,* 2019a). Instead their conversations were dominated by information gathering, questions and persuasion (Bard *et al.,* 2017; Svensson *et al.,* 2019a). Ritter *et al.* (2019) recently demonstrated that dominance in the veterinarian and a high use of information gathering in consultations were associated with a lower stated likelihood by farmers to implement veterinary advice. In a similar vein, persuasion and confrontation are behaviors that have been shown to be negatively associated with behavior change in consultancies (Miller and Moyers, 2017). Hence, veterinarians speaking in this way run the risk of counteracting their purpose to encourage clients to follow veterinary recommendations.

One change-orientated evidence-based communication methodology being increasingly implemented across numerous sectors is motivational interviewing (**MI**; Miller and Moyers, 2017). This methodology was developed in alcohol abuse consultations and has successfully been used to reduce tobacco and drug use and to promote positive lifestyle changes (Hettema *et al.,* 2005; Lundahl *et al.,* 2010). Lately, the MI methodology has also been found to be a helpful tool in enforcement situations for food safety, health safety and environmental inspectors (Forsberg *et al.,* 2014; Wickström *et al.,* 2017) and was rated by cattle veterinarians as highly relevant to their profession (Svensson *et al.,* 2020).

For veterinarians to continue to be effective and valued consultants in animal health, efficiency in their services is of importance. Given the weaknesses demonstrated in veterinarians’ communications skills, adopting a client-centered communication methodology such as MI may be one means to increase efficiency in VHHM services, as suggested by Bard *et al.* (2017) and Svensson *et al.* (2019a). Veterinary herd health management services involve complex consultancies and little is known about the communication style best suited for veterinarians to be efficient. To estimate the potential of MI to facilitate clients’ implementation of preventive measures in VHHM, studies that objectively measure the effect of the methodology on these client behaviors are warranted.

In MI, *Change Talk* is defined as the client’s own statements expressing consideration of, motivation for or commitment to behavior change. Motivational interviewing research uses the amount of *Change Talk* expressed by the client as an outcome measure of communication skills, because it has been shown to be strongly correlated with clients later adopting the behavior change in question (Apodaca and Longabaugh, 2009). Several instruments have been developed to assess client verbal responses in consultations. However, the most valid are time consuming and therefore costly (Martin *et al.,* 2005). A more practical and economically reasonable instrument is the Client Language Easy Rating **(CLEAR)** coding system (Hagen and Moyers, 2012). Client Language Easy Rating assesses and summarizes clients’ responses in three categories: *Change Talk*, *Sustain Talk* (i.e. corresponding statements related to the *status quo)* and *Neutral Talk*. When the amount of *Change Talk* and *Sustain Talk* in a session is all that is of interest, CLEAR is believed to represent an appropriate and efficient way to characterize these types of client language (Hagen and Moyers, 2012).

The aim of the present study was to evaluate the potential of MI to facilitate client behavior change in VHHM by investigating the effect of dairy cattle veterinarians’ MI skills on client *Change Talk* and *Sustain Talk* during VHHM visits. More specifically, the study aimed to test the hypothesis that clients conversing with veterinarians who had greater MI skills would express more *Change Talk* and less *Sustain Talk* than clients conversing with veterinarians who had a lower level of MI skills. Preliminary results from the present study have previously been published in abstract form (Svensson et al., 2019c).

## Material and methods

In total, 36 cattle veterinarians audio-recorded VHHM consultancies on 170 Swedish cattle farms (164 dairy, 4 cow-calf beef and 2 specialized beef) using digital voice recorders. Recordings were made between June 2016 and January 2017 (*n* = 18) or between June 2017 and January 2018 (*n* = 18). Veterinarians wore voice recorders and uploaded recordings to a webpage at the coding laboratory MIC Lab AB, Stockholm (www.miclab.se). Professional coders at MIC Lab AB coded the clients’ *Change Talk* and *Sustain Talk* using the CLEAR coding system. The quality of the recordings varied and was sometimes reduced by sounds from cows, machinery and interrupting telephone calls. The quality, however, was generally acceptable for coding. Each veterinarian was requested to record five consultancies; details about these consultancies have been reported by Svensson *et al.* (2019b).

**Figure 1:**
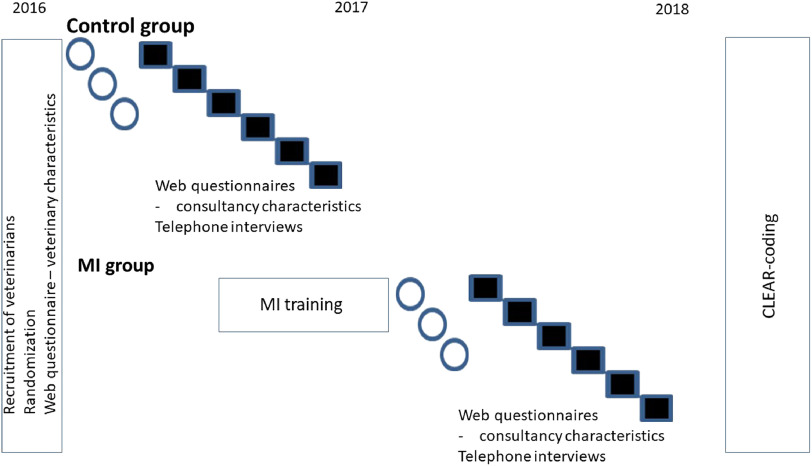
Design of the study investigating effect of veterinary motivational interviewing (MI) skills on client responses in veterinary herd health management conversations on 170 Swedish cattle farms.

Half of the veterinarians (*n* = 18) had participated in a 6-month MI training program between September 2016 and March 2017(before they recorded their conversations); the rest were untrained. Before the consultancies took place, we assessed veterinarians’ MI skills from role-play conversations with professional actors. At the start of the project, the veterinarians had filled in a web questionnaire (https://www.netigate.net/sv/) about their characteristics (Svensson *et al.,* 2019a), from which we received information about their gender and experience in VHHM. Veterinarians had also filled in a web questionnaire about (1) the conditions of their farm visit, (2) their view about the consultation and (3) the outcomes of the consultation. From Part (1) of this questionnaire, we retrieved information about type of visit (pre-defined categories) and the number of participants from the farm; from Part (2) we received information about whether veterinarians felt that they and the client, respectively, had allocated sufficient time to the consultation (Likert scale 1 to 6). After the consultations, clients were interviewed via telephone and data about their age, gender, education, role on the farm, satisfaction with the consultation and if they felt that they and the veterinarian, respectively, had allocated sufficient time to the consultation were collected. The telephone interviews have been further described by Svensson *et al.* (2019b). The study design is outlined in Figure 1.

### Participating veterinarians and farms

The selection of participating veterinarians has been described previously by Svensson *et al.* (2019a). In short, volunteers were selected by the two largest employers of Swedish dairy cattle veterinarians – the District Veterinary Organization (Swedish Board of Agriculture) and the regional dairy associations – or among self-employed dairy cattle practitioners involved in the main Swedish VHHM network. Out of the total number of Swedish dairy cattle veterinarians involved in VHHM (*n* = 97: 56 employed by District Veterinary Organization, 23 by dairy associations and 18 self-employed), 42 veterinarians participated in the project and were randomly distributed into 2 groups (trained MI group and untrained control group). The training, described in detail by Svensson *et al.* (2020), consisted of six workshops with theoretical lectures and practical training. During the time between workshops, participants were to read and reflect on chapters in the main MI handbook by Miller and Rollnick (2012) and to practice their skills. Due to lack of time, four veterinarians terminated their participation in the project before they started their training, one never finished the training and one never recorded any consultancies. Out of the 36 veterinarians included in the present study, there were 2 men and 34 women. Eighteen were district veterinarians, 13 were animal health veterinarians from the regional dairy associations and 5 were self-employed veterinarians. Veterinarians were stationed all over the country, in both intensive farming areas and woodland areas. All 36 veterinarians received MI training without any cost as part of the project. Control veterinarians received training from September 2017 to March 2018 (i.e. after they had finished all their recordings for the present study).

The selection of participating farms has been described previously by Svensson *et al.* (2019b). In short, a convenience sample of farms chosen by the veterinarians from among their clients was included and farmers were informed by the veterinarians about the purpose and design of the project. Clients received no compensation to participate in the study. We asked veterinarians in both groups (trained MI group and untrained control group) to provide the same information about the communication training in the project so that farms would be blinded to whether the veterinarian had received MI training or not. Six farms were visited by two different veterinarians. One farm was visited in the same year by two different veterinarians who were both trained in MI. Recommendations from these two veterinarians dealt with totally different areas (biosecurity and udder health); the biosecurity conversation was considered to have negligible impact on the response talk in the udder health conversation and *vice versa*. Both observations therefore remained in the study. The other five farms were first visited by a control veterinarian and one year later by a veterinarian trained in MI.

### Assessing motivational interviewing skills

Each veterinarian conducted three role-play conversations reflecting *telephone consultations with a client whom the veterinarian previously had met on the farm when the time had been restricted and an agreement therefore had been made to continue and finish the discussion over the telephone*. The role-plays were designed to provide controlled conditions for veterinarians to demonstrate relevant MI skills. For reference, veterinarians’ MI skills were also assessed from the 170 audio-recorded on-farm VHHM consultancies mentioned above. Veterinarians’ MI skills were assessed using the Motivational Interviewing Treatment Integrity coding system 4.2.1 (**MITI**; Moyers *et al.*, 2014). The MITI identifies frequency counts of 10 verbal behaviors as well as assessments of 4 global scores on a Likert scale ranging from 1 (‘low’) to 5 (‘high’) based on 20 min of a conversation. The coding manual also specifies six summary measurements derived from the 14 original variables (Moyers *et al.*, 2014). The role-plays and MITI codings (coded by MIC Lab AB) have previously been described in detail by Svensson *et al.* (2019a and 2020).

Based on the MITI coding results, we categorized veterinarians’ MI skills as ‘poor’, ‘near moderate’ and ‘moderate’. We further sub-categorized ‘poor’ skills into ‘poor_untrained’ and ‘poor_trained’, because differences between untrained and trained veterinarians in MI skills other than those expressed by the MITI variables could not be excluded. In order to categorize in this way, we used the summary MITI variables *Relational* and *MI-non-adherent behaviors* and the original MITI variable *Cultivating Change Talk*. The MITI variable *Relational* was calculated as (*Partnership* + *Empathy*)/2, where *Partnership* expressed the extent to which the advisor actively fostered collaboration and power sharing with the client, and *Empathy* was how the advisor understood or made an active effort to grasp the client’s perspective and experience. *MI-non-adherent behaviors* were *Persuade* (overt attempts to change a client’s opinions, attitudes or behaviors using tools such as logic, compelling arguments, self-disclosure, facts, biased information, advice, suggestions, tips, opinions or solutions to problems) and *Confront* (directly and unambiguously disagreeing, arguing, correcting, shaming, blaming, criticizing, labeling, warning, moralizing, ridiculing or questioning a client’s honesty). *Cultivating Change Talk* expressed the extent to which the advisor actively encouraged the client’s own language in favor of the behavior change goal as well as the client’s confidence to make the change. Because information about client ambivalence was lacking for the on-farm VHHM consultancies, we did not use *MI-non-adherent behaviors* in the reference categorization based on the on-farm recordings but used only *Relational* and *Cultivating Change Talk.*

We used the following thresholds to define the veterinarians who reached ‘near moderate’ and ‘moderate’ competency: ‘moderate’ competency – *Relational* ⩾3.5, *Cultivating Change Talk* ⩾3 and *MI-non-adherent behaviors* ⩽2; ‘near moderate’ competency – *Relational* ⩾3.5, *Cultivating Change Talk* ⩾2.7 and *MI-non-adherent behaviors* <4. Veterinarians who did not reach these thresholds were categorized as ‘poor’. In the reference categorization based on the on-farm recordings, thresholds were ‘moderate’ competency – *Relational* or *Empathy* ⩾3 and *Cultivating Change Talk* ⩾2; ‘poor’ competency – *Relational* or *Empathy* ⩽2 or *Cultivating Change Talk* ⩽1.2. Veterinarians who did not meet these thresholds were categorized as ‘near moderate’. Thresholds were chosen based on MI literature and experience of MITI coding of conversations in different contexts. *Relational* ⩾3.5 and *Cultivating Change Talk* ⩾3 are thresholds suggested in the MITI manual (Moyers *et al.*, 2014). We deleted one veterinarian who only had one recorded VHHM visit from the reference categorization based on on-farm recordings, as one recording was not considered sufficient to give a reliable measurement.

### Assessing client Change Talk

Three coders performed all CLEAR codings of the 170 audio-recordings from on-farm VHHM consultations according to the CLEAR manual, translated to Swedish (Hagen and Moyers, 2012). The professional coders at MIC Lab AB perform MITI codings continuously and had been trained in CLEAR coding before the present study. To sustain coders’ competence, coders at MIC Lab AB participate in a quality assurance program. The program comprises weekly training sessions based on independently coded recordings. Coders also discuss especially difficult coding sessions between themselves regularly. Further information about the quality assurance program is provided in Supplementary Material S1. The CLEAR manual specifies frequency counts of two main categories of client talk, *Change Talk* and *Sustain Talk*, each comprising seven sub-categories. *Change Talk* comprises the sub-categories *Desire to change, Ability to change, Reason to change, Need to change, Commitment to change, Taking steps towards change* and *Other Change Talk*. The seven sub-categories of *Sustain Talk* are *Desire not to change, Ability not to change, Reason not to change, Need to not change, Commitment not to change, Taking steps away from change and Other Sustain Talk*. We summarized client responses as *Change Talk* and *Sustain Talk*. We also calculated another outcome variable, *Proportion of Change Talk*, defined as *Change Talk* frequency over the sum of *Change Talk* frequency plus *Sustain Talk* frequency (%*Change Talk* = *Change Talk*/(*Change Talk* + *Sustain Talk*)).

Coders started CLEAR coding the on-farm VHHM recordings when all veterinarians (from both groups) had recorded all consultancies. The order in which coders coded the recordings was randomized so that consultancies from both MI-trained veterinarians and untrained control veterinarians were coded in parallel. Consultations were encrypted during uploading to the web page and registered in a database at a protected server. Coders did not know the identities of veterinarians nor their group. For reliability reasons, the MITI recommends to code 20 min of each consultation. Because recordings were used both for MITI and CLEAR coding, we coded 20 min of each consultation. Veterinarians were instructed to record a minimum of 20 min consultation on each farm and to select the time period during which they were consulting the client about any behavior change (i.e. implementation of preventive measures). However, 21 recordings (all included in the present study) were shorter (10 to 17 min). If veterinarians recorded longer consultations, we coded those parts indicated by the veterinarians to be about behavior change. When veterinarians had indicated longer sequences than 20 min as relevant, we chose random sequences of 20 min to code. Veterinarians were not specifically informed that the audio-recordings would be subjected to CLEAR coding.

### Data editing and statistical analyses

Descriptive statistics of *Change Talk* and *Sustain Talk* were calculated using Microsoft Excel (Microsoft Corp., Redmond, WA, USA). The frequencies of *Change* and *Sustain Talk* for conversations shorter than 20 min were adjusted to 20 min by multiplying the frequency with 20/(number of minutes of the recordings).We investigated the effect of MI skills on client response talk using three cross-classified regression models. Two Poisson regression models, with random intercepts for veterinarian and client (farm) and offset for number of minutes of the recordings, were estimated in the statistical software R (Version 3.5.3., R Core Team 2019, https://www.R-project.org/) using the package glmmTMB (R package version 0.2.3. Brooks *et al.,* 2017) for the two response variables *Change Talk* and *Sustain Talk*. The offset in models standardizes the response variable to the length of the recording, thus, in our case, making the rate of the different types of client speech the modeled response. A logistic regression model with the same random intercepts but with the response variable *Proportion of Change Talk* was also estimated using the same package. The effects of the following extra explanatory variables were assessed: gender, VHHM experience and type of veterinarian, age, education and role of the client, if both client and veterinarian felt that the time allocated for the consultancy was sufficient, if the gender of the client and veterinarian were the same (gender concordance), number of participants from the farm, visit type and the client’s satisfaction with the consultation. Interactions and sequence of veterinarians’ visits (time within veterinarian) were not investigated because of the limited number of observations. The R code is provided in Supplementary Material S2. All extra explanatory variables except age of the veterinarian were categorical; categories of each variable are shown in Table 1. Animal health veterinarians worked with preventive medicine only, whereas general large animal practitioners also made treatment visits. Lower education was defined as primary or secondary level of education and higher education as tertiary level education. The variable *Sufficient time* was created from the responses (Likert scale 1 to 6) by the veterinarian in the web questionnaire (own and the client’s time) and by the client in the telephone interview (own and veterinarian’s time) so that *Sufficient time* was classified as ‘no’ when either the veterinarian or the client rated a time variable below four and as ‘yes’ in all other cases. The client was denoted as satisfied with the consultation if she or he had rated the satisfaction with both the veterinarian’s behavior and competency (Likert scale 1 to 6) as more than three or the sum of the two ratings was eight or more.

**Table 1:** Results from multivariable Poisson regression model^a^ of the associations between veterinarians’ (*n* = 36) skills in motivational interviewing (MI) and rate of client Change Talk in 170 veterinary herd health management (VHHM) consultations on Swedish cattle farms

				Multiplicative effect		
Level of observation	Parameter	Level	Number	Estimate; 95% CI^b^	*P*	Overall *P* ^c^
Veterinarian	MI skills	Poor_untrained	18	Ref		0.06
		Poor_trained	6	0.99; 0.70 to 1.40	0.97	
		Near moderate	6	1.07; 0.77 to 1.50	0.68	
		Moderate	6	1.55; 1.12 to 2.10	<0.01	
	Gender	Male	2	Ref		
		Female	32	0.91; 0.61 to 1.40	0.67	
	VHHM experience	⩽5 years	22	Ref		
		>5 years	14	1.08; 0.85 to 1.40	0.53	
	Vet type	Animal health vet	13	Ref		
		General practitioner	23	0.89; 0.69 to 1.10	0.35	
	Gender concordance	No	124	Ref		
		Yes	46	1.20; 0.98 to 1.50	0.08	
Consultancy	Sufficient time	No	34	Ref		
		Yes	136	1.04; 0.83 to 1.30	0.73	
	Number of clients	One	150	Ref		
		Multiple	20	0.99; 0.72 to 1.30	0.94	
	Visit type	Strategic	38	Ref		0.06
		Herd health problem	30	0.69; 0.50 to 0.95	0.02	
		Other	102	0.81; 0.63 to 1.00	0.07	
Client	Age	Continuous (decades)		1.01; 0.93 to 1.10	0.79	
	Education	Lower	104	Ref		
		Higher	66	0.96; 0.79 to 1.20	0.64	
	Role	Owner	133	Ref		
		Employee	37	1.00; 0.78 to 1.30	0.99	
	Satisfied	No	2	Ref		
	with consultancy	Yes	168	1.73; 0.65 to 4.60	0.27	

a SD of random intercept of veterinarian: 0.21 (standard error (SE): 0.079) and client (farm): 0.38 (SE: 0.052).

b 95% confidence interval.

c Overall *P*-value for chi-square test for variables with more than two categories.

For each model, randomized quantile residuals were obtained by the R package DHARMa (R package version 0.2.4. https://CRAN.R-project.org/package=DHARMa) and assessed graphically and with tests of residual distribution, together with tests of under- and overdispersion and zero inflation. None of the models showed any clear visual deviation for the residual distribution from the assumed error distribution, and none of the tests of deviations from typical model misspecifications indicated any problems. Multicollinearity was assessed for each model with generalized variance inflation factor, due to the presence of categorical explanatory variables. No evidence of multicollinearity was found, with a rule of thumb threshold at three which corresponded to an ordinary variance inflation factor of nine. Results from the model validation are shown in Supplementary Material S3.

## Results

The frequency count of *Change Talk* per 20 min ranged from 0 to 18 (median: 6; interquartile range: 4 to 8) and of *Sustain Talk* from 0 to 13 (median: 2; interquartile range: 1 to 4). Distribution of characteristics of veterinarians, clients and consultancies is shown in Table 1. The age of clients ranged from 20 to 74 (median: 49; interquartile range: 38 to 56) years; 91 were men and 64 were women, whereas both genders were represented in 15 of the conversations with multiple clients. Clients were overall highly satisfied with their veterinarians. On the 170 farms, all but 10 (94%) clients scored satisfaction with the veterinarian’s attitude at 5 or 6 (range: 3 to 6; median: 6; interquartile range: 5 to 6) and all but 14 (92%) stated their satisfaction with the veterinarian’s competency to be 5 or 6 (range: 1 to 6; median: 5; interquartile range: 5 to 6). None of the untrained control veterinarians reached MI skills comparable to the thresholds set to categorize ‘near moderate’ competency (i.e. the MI skills of all untrained veterinarians were categorized as poor_untrained). Of the trained veterinarians, six reached ‘moderate’ skill, six ‘near moderate’ skills and six were categorized as having ‘poor’ skills (Table 1). Before training, none of the trained veterinarians had reached ‘near moderate’ skills.

Table 1 also presents the results from the cross-classified model investigating associations with *Change Talk*. The veterinarian’s MI skills were associated with the client’s *Change Talk*, with clients speaking to veterinarians that had reached ‘moderate’ skills expressing 1.6 times more *Change Talk* (*P* = 0.008) than clients speaking to untrained veterinarians. Results regarding *Sustain Talk* and *Proportion of Change Talk* were inconclusive (Tables 2 and 3). Clients of animal health veterinarians expressed more *Sustain Talk* (*P* = 0.003; Table 2) and a lower *Proportion of Change Talk* (*P* = 0.01; Table 3) than clients of general large animal practitioners. There was 1.2 times more *Change Talk* in conversations with clients of the same gender as the veterinarian compared to conversations without gender concordance, but the confidence interval **(CI)** was 0.98 to 1.50. The multiplicative effects of *Change Talk* in conversations from visits regarding herd health problems or visits of other types (as compared to strategic visits) were 0.69 and 0.81, but CIs were 0.50 to 0.95 and 0.63 to 1.00, respectively (Table 1). The odds ratio for the *Proportion of Change* Talk for clients satisfied with the conversation (as compared to for unsatisfied clients) was 2.8, but CI was 0.95 to 8.40 (Table 3). Using the veterinarian’s MI skills based on on-farm conversations rather than on role-play conversations in the multivariable analyses gave the same results (results not shown).

**Table 2:** Results from a multivariable Poisson regression model^a^ of the associations between veterinarians’ (*n* = 36) skills in motivational interviewing (MI) and rate of client Sustain Talk in 170 veterinary herd health management (VHHM) consultancies on Swedish cattle farms

				Multiplicative effect		
Level of observation	Parameter	Level	Number	Estimate; 95% CI^b^	*P*	Overall *P* ^c^
Veterinarian	MI skills	Poor_untrained	18	Ref		0.51
		Poor_trained	6	1.08; 0.71 to 1.60	0.73	
		Near moderate	6	1.05; 0.72 to 1.50	0.80	
		Moderate	6	1.35; 0.92 to 2.00	0.13	
	Gender	Male	2	Ref		Ref
		Female	32	1.18; 0.72 to 1.90	0.52	
	VHHM experience	⩽5 years	22	Ref		Ref
		>5 years	14	1.17; 0.88 to 1.60	0.28	
	Vet type	Animal health vet	13	Ref		Ref
		General practitioner	23	0.64; 0.47 to 0.86	<0.01	
	Gender concordance	No	124	Ref		Ref
		Yes	46	1.20; 0.89 to 1.60	0.23	
Consultancy	Sufficient time	No	34	Ref		Ref
		Yes	136	0.84; 0.61 to 1.20	0.28	
	Number of clients	One	150	Ref		Ref
		Multiple	20	0.90; 0.59 to 1.40	0.62	
	Visit type	Strategic	38	Ref		0.22
		Herd health problem	30	0.67; 0.42 to 1.10	0.08	
		Other	102	0.84; 0.61 to 1.20	0.29	
Client	Age	Continuous (decades)		1.12; 0.98 to 1.30	0.09	
	Education	Lower	104	Ref		Ref
		Higher	66	1.15; 0.88 to 1.50	0.31	
	Role	Owner	133	Ref		Ref
		Employee	37	1.23; 0.87 to 1.80	0.24	
	Satisfied	No	2	Ref		Ref
	with consultancy	Yes	168	0.65; 0.22 to 2.00	0.45	

a SD of random intercept of veterinarian: 0.08(SE: 0.26) and client (farm): 0.55 (SE: 0.078).

b 95% confidenceinterval.

c Overall *P*-value for chi-square test for variables with more than two categories.

**Table 3:** Results from a multivariable logistic regression model^a^ of the associations between veterinarians’ (*n* = 36) skills (*n* = 36) in motivational interviewing (MI) and Proportion of Change Talk in 170 veterinary herd health management (VHHM) consultations on Swedish cattle farms

				Proportion of Change Talk		
				Odds ratio		
Level of observation	Parameter	Level	Number	Estimate; 95% CI^b^	*P*	Overall *P* ^c^
Veterinarian	MI skills	Poor_untrained	18	Ref		0.88
		Poor_trained	6	0.93; 0.63 to 1.40	0.72	
		Near moderate	6	1.02; 0.79 to 1.40	0.93	
		Moderate	6	1.12; 0.79 to 1.60	0.51	
	Gender	Male	2			
		Female	32	0.90; 0.56 to 1.40	0.65	
	VHHM experience	⩽5 years	22			
		>5 years	14	0.89; 0.68 to 1.20	0.38	
	Vet type	Animal health vet	13			
		General practitioner	23	1.44; 1.09 to 1.90	0.01	
	Gender concordance	No	124			
		Yes	46	1.02; 0.77 to 1.40	0.87	
Consultancy	Sufficient time	No	34			
		Yes	136	1.26; 0.93 to 1.70	0.14	
	Number of clients	One	150			
		Multiple	20	1.03; 0.68 to 1.60	0.89	
	Visit type	Strategic	38	Ref		0.90
		Herd health problem	30	1.07; 0.69 to 1.70	0.75	
		Other	102	0.98; 0.73 to 1.30	0.88	
Client	Age	Continuous (decades)		0.89; 0.79 to 1.00	0.06	
	Education	Lower	104			
		Higher	66	0.86; 0.66 to 1.10	0.25	
	Role	Owner	133			
		Employee	37	0.80; 0.58 to 1.10	0.18	
	Satisfied	No	2			
	with consultancy	Yes	168	2.82; 0.95 to 8.40	0.06	

a SD of random intercept of veterinarian: <0.001 (SE: 0.44) and client (farm): 0.24 (SE: 0.12).

b 95% confidence interval.

c Overall *P*-value for chi-square test for variables with more than two categories.

## Discussion

Veterinarians’ MI skills were associated with client *Change Talk,* but results regarding *Sustain Talk* and *Proportion of Change Talk* were inconclusive. Previous studies have demonstrated associations between MI skills and all three variables. Magill *et al.* (2018) reported from a meta-analysis of 36 studies that MI skills of non-veterinary consultants in interventions targeting a range of behavioral outcomes (alcohol use, drug use, gambling, diet, exercise and medical adherence) were positively associated with both *Change Talk* and *Sustain Talk*. However, on average, improved MI skills were associated with more *Change Talk* rather than *Sustain Talk*. This is consistent with the method of MI, which explores ambivalence and, as the conversation continues, helps the client to resolve this ambivalence into commitment to change (Miller and Rollnick, 2012). A link between *Change Talk* and behavior change at follow-up has been demonstrated in several studies (Amrhein *et al.,* 2003; Moyers *et al.,* 2009; Pirlott *et al.,* 2012), and a systematic review of studies found that *Change Talk* was consistently related to positive client outcome (Romano and Peters, 2016). This highlights the importance of the findings in the present study and demonstrates an indirect link to outcome of VHHM consultancies suggesting that learning to practice MI may be one means to increase efficiency of veterinary services.

It is unclear what level of MI fidelity is ‘good enough’ to facilitate change within particular contexts and thus the level of MI skills a veterinarian should have to get results. In the present study, we categorized veterinarians’ MI skills based on both relational and technical skills (the MITI variables *Relational*, *MI-non-adherent behaviors* and *Cultivating Change Talk*). These variables were chosen because the skill of empathy has been positively associated and *MI-non-adherent behaviors* negatively associated with outcome. The technical skill *Cultivating Change Talk* has been positively associated with *Change Talk* (Lindqvist *et al.,* 2017). For role-play conversations, the thresholds for *Relational* and *Cultivating Change Talk* were based on those suggested to represent ‘fair competency’ in the MITI manual (Moyers *et al.,* 2014). Although firm suggestions are lacking with regard to MI-non-adherent behaviors, it is generally recognized that this type of speech should ideally not occur in MI consultations. Coding is difficult, and because the veterinary context was new to the coders before we trained them for the present study, they may have misinterpreted some situations and miscoded speech as MI-non-adherent. To account for this, we chose ⩽2 as a threshold for ‘moderate’ skills for this variable. Thresholds for ‘near moderate’ skills in role-play conversations and for the on-farm conversations were chosen based on experience of MITI coding of conversations in different contexts. In the on-farm conversations, none of the veterinarians reached the threshold used for the role-play conversations. Although previous studies have demonstrated associations between MI skills and outcome, research has not yet been able to specify clear thresholds (Magill *et al.,* 2018). A definition of ‘moderate’ MI skills was associated with *Change Talk,* but results regarding ‘near moderate’ or ‘poor’ skills were inconclusive. This may indicate that a certain level of MI skills is needed to have an impact. Further studies are needed to explore the most suitable thresholds to define various levels of MI skills in the veterinary profession.

Svensson *et al.* (2020) demonstrated that cattle veterinarians were able to reach ‘moderate’ MI skills from a 6-month training program consisting of 6 days of workshops separated by period of literature studies and practical training of their new skills. However, the majority of participating veterinarians in this study did not reach this level of skills, highlighting the challenges of teaching MI methodology and the need for sufficient practice. Motivational interviewing takes time to learn and to maintain, and it may not be possible to fit sufficient practice into the every-day-work of a cattle practitioner.

There was a higher rate of *Sustain Talk* and lower *Proportion of Change Talk* in consultations with animal health veterinarians compared to in those with a general large animal practitioner. This finding is difficult to explain but may be due to animal health veterinarians being more tempted to use their expertise and suggest actions to their clients (MITI variable *Persuade*). Animal health veterinarians generally have larger volumes of VHHM services in their work compared to general large animal practitioners and may have been more confident in their advisory role. Confidence may be built not only from longer experience as veterinarians or years in VHHM but also from larger volumes of VHHM. Svensson *et al.* (2019a) found that veterinarians with more years in practice had lower *Relational* scores and expressed more persuasion than those with more recent veterinary degrees. In line with that finding, Svensson *et al.* (2020) reported that veterinarians with longer experience in VHHM did not improve in practicing *Cultivating Change Talk* after their MI training.

The variable with largest average effect on proportion of *Change Talk* was client satisfaction (odds ratio: 2.8; CI: 0.95 to 8.40). Ritter *et al.* (2019) previously suggested client satisfaction to be a proxy for farmers’ preparedness to adopt veterinary advice. Just as in the study by Ritter *et al.* (2019), clients in the present study were highly satisfied with their veterinarian. In fact, only two clients stated they were unsatisfied; hence, these results should be interpreted with caution.

The results of the present study suggest that the association between *Change Talk* and type of visit should be further evaluated in future studies. Strategic visits aim to optimize animal health and production in a longer perspective, and it may be more logical to discuss farm goals in this type of consultations compared to on VHHM visits initiated as a consequence of specific herd health problems and other advisory visits. The focus on farm goals may improve veterinarian–client relations and trust that in turn may render clients to view their relationship with the veterinarian more positively and to adopt veterinary advice, as indicated by findings by Svensson *et al.* (2019b) and Bard *et al.* (2019).

### Methodological considerations

We chose to assess veterinarians’ MI skills based on role-play with professional actors. This approach was chosen as role-play methodology has shown promise in comparison with using real clients (Imel *et al.,* 2014) and our previous work suggested veterinarians’ communication patterns between role-play and real contexts were stylistically similar (Svensson *et al.* 2019a). Additionally, this approach standardized the conditions for MI communication, allowing for reliable categorization of veterinarians in terms of estimating their MI skills. The role-plays were designed and the actors were trained to provide controlled conditions for participants to demonstrate all their relevant MI skills; consultations had clear behavior targets and actor clients had ambivalent perceptions. To ensure methodological validity, we also assessed how veterinarians would have been categorized based on performance within the same on-farm consultations from which client CLEAR coding data were drawn. Minor differences were found in overall skills categorization, but this method provided the same associations with outcome variables (results available on request) indicating the basis for categorization of MI skills was not critical to these results. Further research is needed to explore if more nuanced differences may exist between such sample groups.

Because of its exploratory nature, multiple testing issues have not been considered in the present study. Observed effects should be verified in future studies and until then interpreted with caution. The limited spread in MI skills among veterinarians (few veterinarians reached ‘moderate’ skills, and none reached higher levels of MI skills) may have reduced the power of this study, making it less possible to identify associations with client responses. Future research using samples with larger variation may be used to verify the present results and to find further associations between veterinarians’ MI skills and client responses.

Also, we were unable to use one of the most accurate coding instruments, the Motivational Interviewing Sequential Code for Observing Process Exchanges (**SCOPE**; Martin *et al.,* 2005), to assess client response. The SCOPE requires that recorded consultations are transcribed and that coders go through the recordings twice to assess each client utterance against one of 16 client codes. In contrast, CLEAR coding does not require a transcript and the coder only needs to listen to the recorded conversation once. Client Language Easy Rating does not code global ratings but only counts of *Change Talk, Sustain Talk* and *Neutral Talk.* Client Language Easy Rating coding is also not sequential, so behaviors are coded using only tallies. Future studies with larger budgets enabling more precise methods may reveal more associations. Furthermore, qualitative methodologies may complement quantitative efforts such as the present study, offering nuanced and in-depth insight into how veterinarians and farmers understand and experience these MI advisory consultations in the VHHM sphere.

Information about coder was not available and the effect of coder could not be included in the statistical models. However, to sustain coders’ competence, coders at MIC Lab AB participated in a quality assurance program. Furthermore, codings were performed in a randomized order, which was likely to reduce further any effects of coder. It is therefore unlikely that the results were biased due to systematic differences between coders. We chose to include a random effect of client (farm) as multiple veterinarians occasionally visited the same farm. Inclusion of a random client (farm) effect even though most farms were only visited once is also a common remedy against so-called overdispersion (i.e. excess variation that is not described by the standard Poisson or logistic regression model). For all models, the variance of the random effect of client (farm) was substantially larger than the variance of the random effect of veterinarian, indicating a larger unexplained variation between clients (farms) than between veterinarians (see also Supplementary Materials S4 and S5). A discussion on potential bias related to the veterinarians’ selection of recordings for coding is presented in Supplementary Material S1.

Clients were a convenience sample selected by the veterinarians from among their customers. Many of the veterinarians had difficulty finding five farms where they could record a 20-min advisory conversation for the study. However, when more farms were available, it is likely that clients perceived by the veterinarians as more satisfied with their services would have had a higher chance of being selected. It is therefore not unlikely that the present study may have overestimated the level of satisfaction by clients and that this may have resulted in higher counts of *Change Talk*. A bias in the effect of MI skill on client response talk in these data is not anticipated because the same sampling method was used by all veterinarians to select clients.

The participating veterinarians were not from a random sample, but most likely represented cattle veterinarians most interested in communication and advisory services. Participants were randomized into the two groups and we also controlled for factors that may have been unequally distributed in spite of the randomization (type of veterinarian, gender, VHHM experience and type of visits) in the cross-classified analyses. Coders did not know the identity or the group of veterinarian, and codings for both groups (trained MI-veterinarians and untrained control veterinarians) were made in parallel and in a randomized order. Veterinarians from both groups were instructed to provide the same information to the farms so that clients would be unaware if their veterinarian was trained or untrained. This approach should merit valid comparisons.

## Conclusions

To conclude, in this exploratory study we identified an association between veterinarians’ MI skills and client *Change Talk*, a variable known to be correlated with clients’ adopting of behavior change. The results suggest that MI may be a valuable methodology in VHHM as these services largely focus on changing management routines on farms. Learning to practice MI may be one means to improve adherence to veterinary recommendations and to improve efficiency in VHHM services.

## Software and data repository resources

None of the data were deposited in an official repository.

## Supplementary material

For supplementary material accompanying this paper visit http://dx.doi.org/10.1017/S175173112000107X. click here to view supplementary material
