# Supplementary material for: Dairy veterinarians’ skills in motivational interviewing are linked to client verbal behavior
Source: Animal. 2020 May 19;14(10):2167–77. doi: 10.1017/S175173112000107X (PMC7503130; doi:10.1017/S175173112000107X)
Supplement: Supplementary file 1 [file S175173112000107Xsup001.pdf]

Dairy veterinarians' skills in motivational interviewing are linked to client verbal behavior

C. Svensson, L. Forsberg, U. Emanuelson, K.K. Reyher, A.M. Bard, S. Betnér, C. von Brömssen, and H. Wickström

Animal journal

Supplementary Material S1 – Client Language Easy Rating coding

The MIC Lab AB quality assurance program involved coding using both the Motivational Interviewing Treatment Integrity coding system **(MITI)** and the Client Language Easy Rating coding system **(CLEAR).** Inter-rater reliability between coders regarding MITI codings were calculated and checked twice a year as part of the program; in June 2017 and June 2018, intra-class correlations of the different MITI variables were 0.61-0.97 and 0.52-0.93, respectively. Coders generally perform more MITI than CLEAR coding. Although CLEAR coding was done intensively during the course of this study (April and June 2018) and the quality assurance program dealt with both types of coding in a similar way, inter-rater reliability was never calculated for CLEAR codings.

It was crucial to this study that we coded the parts of the conversations when veterinarians were consulting clients about any behavior change (implementation of preventive measures). To instruct veterinarians to select these parts of the conversation for coding therefore seemed the most reasonable method. In theory, this approach may have allowed trained veterinarians to submit sections when their clients expressed the most *Change Talk*. However, we doubt that this occurred to any considerable extent in practice given:

i) we did not specifically inform participants that these audio-recordings were going to be CLEAR coded;
ii) to recognize and note when the client expressed the most amount of *Change Talk* would have required a very high level of multitasking when the veterinarians were occupied advising their clients;
iii) we know from other parts of the same project and general knowledge about veterinary work that many advisory conversations did not deal with implementation of preventive measures for very long periods, meaning veterinarians therefore often did not have more than one set of 20-minute conversations to choose from;
iv) if veterinarians indeed selected sections with the most *Change Talk*, all trained groups had the same opportunity to do so. Hence, if the observed effect was due to this selection procedure only, all trained groups should have performed better than the 'poor_untrained' group. This was not the case.

Supplementary Material S2 – R code for models

Change Talk Model

```
glmmTMB(data = dataset, formula = change_talk ~ offset(log(minutes)) + age + rp_mi_skills
+ concordance + vet_gender + education + role + sufficient_time + satisfaction +
years_in_vhhm + vet_type + multiplepartner + visit_type + (1|farm)+(1|vet), family = poisson,
REML = TRUE)
```

Sustain Talk Model

```
glmmTMB(data = dataset, formula = sustain_talk ~ offset(log(minutes)) + age + rp_mi_skills
+ concordance + vet_gender + education + role + sufficient_time + satisfaction +
years_in_vhhm + vet_type + multiplepartner + visit_type + (1|farm)+(1|vet), family = poisson,
REML = TRUE)
```

Proportion *Change Talk* Model

```
glmmTMB(data = dataset, formula = cbind(change_talk, sustain_talk) ~ age + rp_mi_skills +
concordance + vet_gender + education + role + sufficient_time + satisfaction +
years_in_vhhm + vet_type + multiplepartner + visit_type + (1|farm)+(1|vet), family =
binomial, REML = TRUE)
```

rp_mi_skills = role play motivational interviewing skills
VHHM = veterinary herd health management
vet = veterinarian

Supplementary Material S3 – Results from model validation

***Change Talk* Model**

Quantile-quantile **(QQ)** plot and residual versus unconditional (on random effects) predictions plot

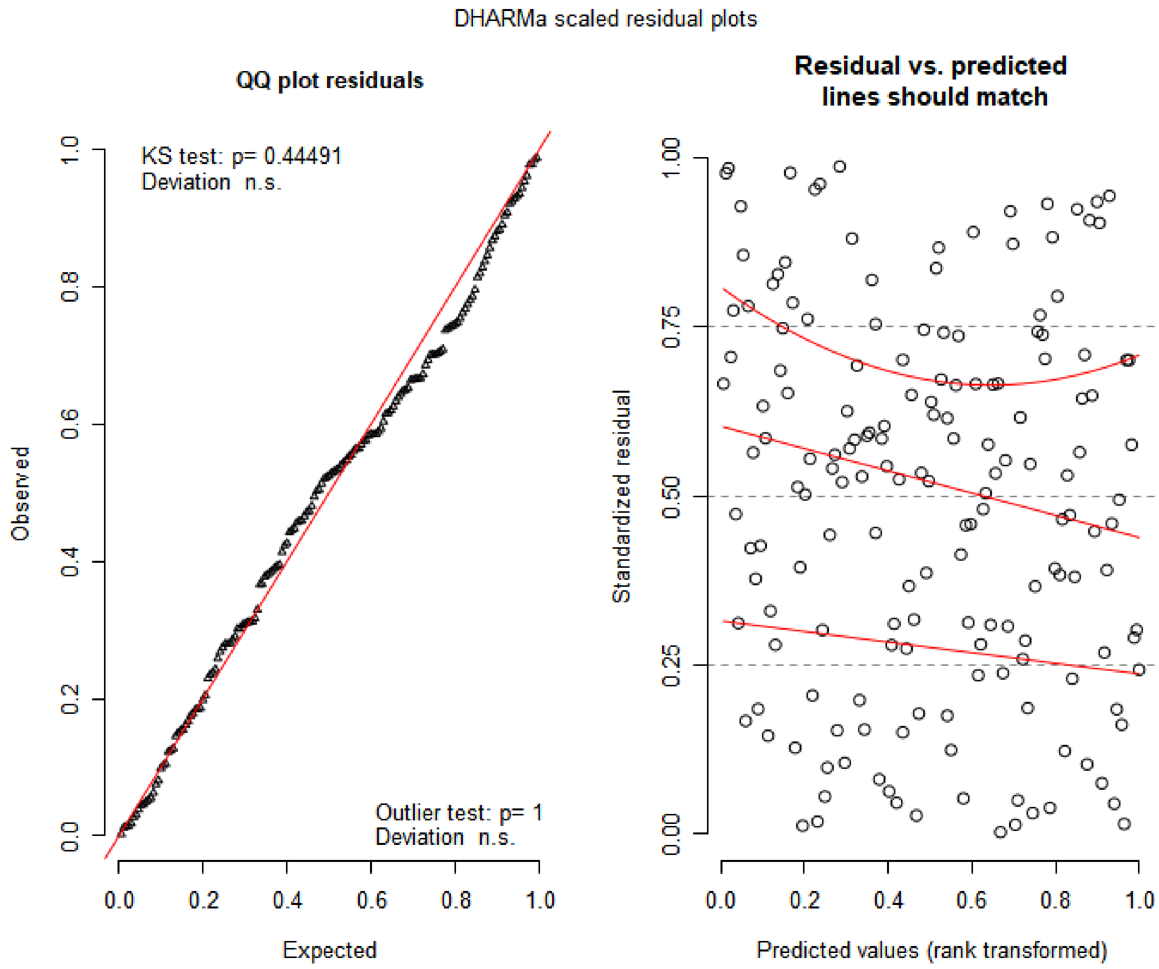

Dispersion test

Estimated dispersion: 0.84, p-value 0.066 (H_0: dispersion = 1, H_A: dispersion ≠ 1)

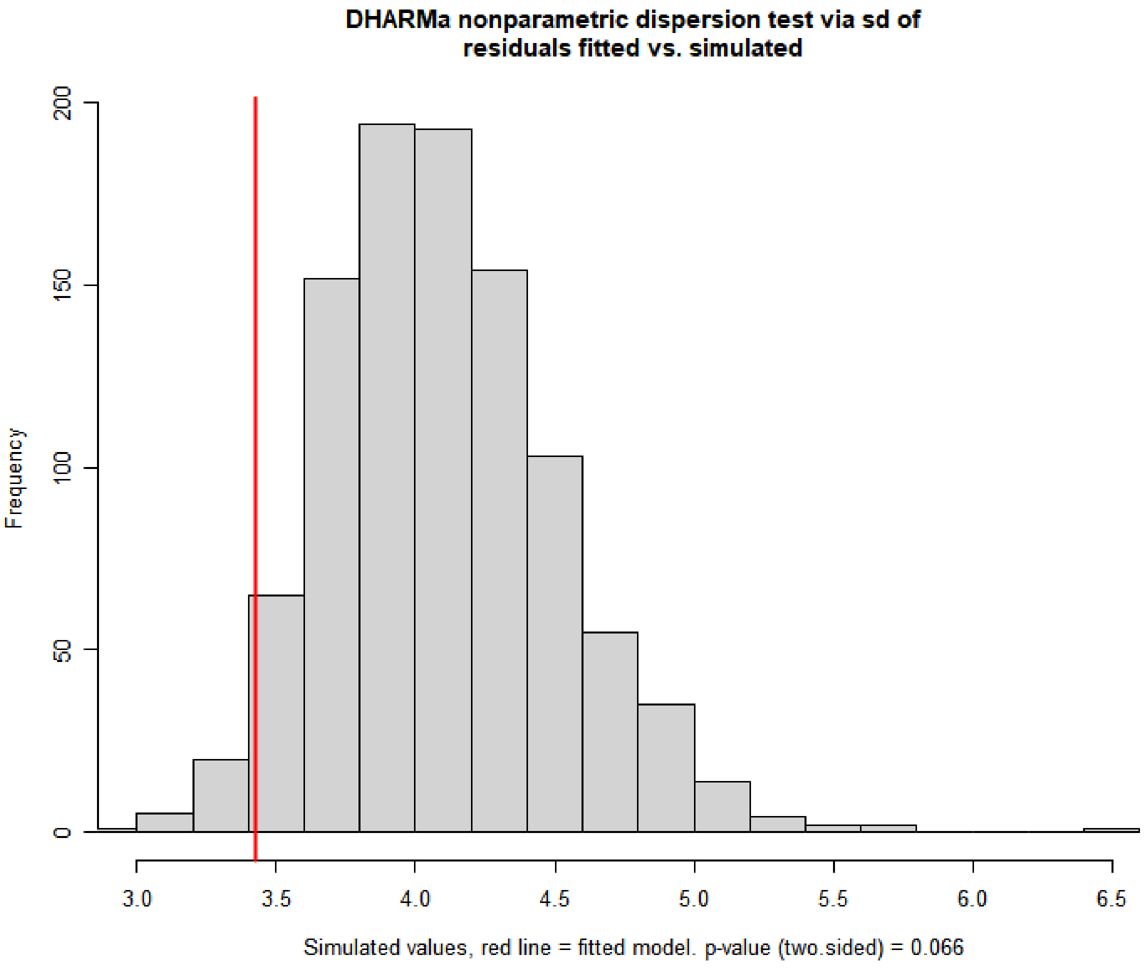

Generalized Variation Inflation Factor **(GVIF)**

Variable	GVIF	Df	$GVIF^{\frac{1}{2Df}}$
age	1.266	1	1.125
rp_mi_skills	1.713	3	1.094
concordance	1.361	1	1.166
vet_gender	1.255	1	1.121
education	1.141	1	1.068
role	1.395	1	1.181
sufficient_time	1.104	1	1.050
satisfaction	1.059	1	1.029
years_in_vhhm	1.164	1	1.079
vet_type	1.288	1	1.135
multiplepartner	1.151	1	1.073
visit_type	1.311	2	1.070

Df = degrees of freedom

***Sustain Talk* Model**

QQ plot and residual versus unconditional (on random effects) predictions plot

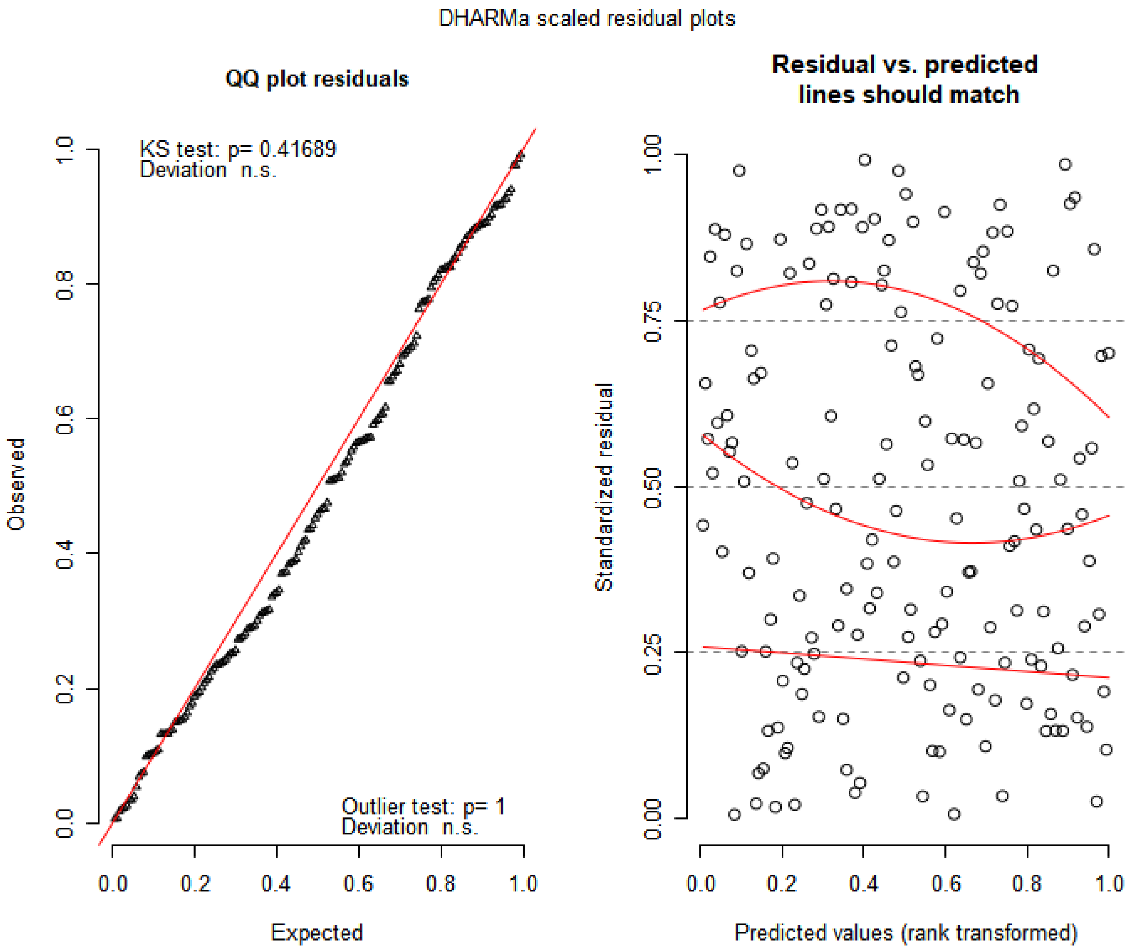

Kolmogorov-Smirnov **(KS)** test

Dispersion test

Estimated dispersion: 0.86, p-value 0.156 (H_0: dispersion = 1, H_A: dispersion ≠ 1)

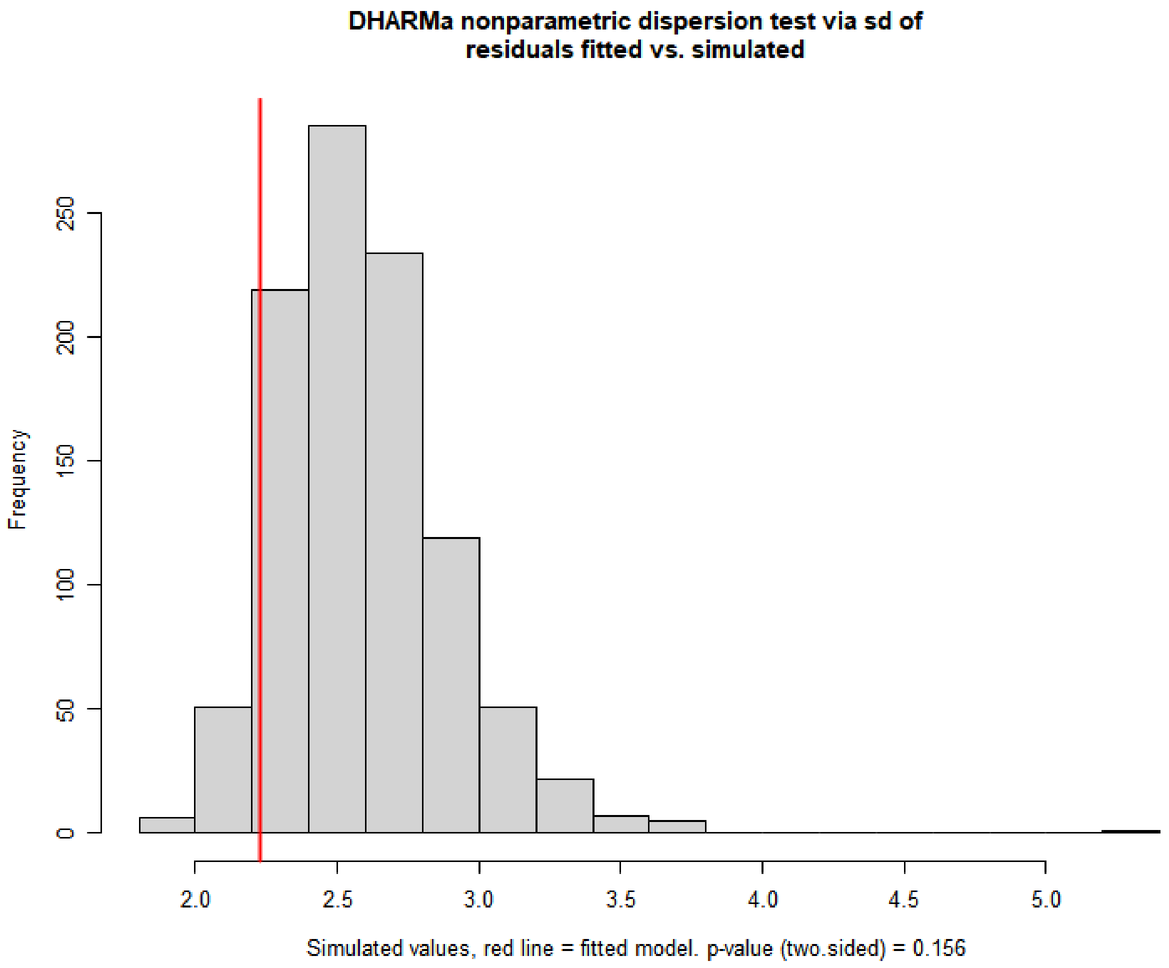

Generalized Variation Inflation Factor

Variable	GVIF	Df	$GVIF^{\frac{1}{2Df}}$
age	1.260	1	1.123
rp_mi_skills	1.755	3	1.098
concordance	1.379	1	1.174
vet_gender	1.264	1	1.124
education	1.139	1	1.067
role	1.384	1	1.176
sufficient_time	1.135	1	1.065
satisfaction	1.103	1	1.050
years_in_vhhm	1.159	1	1.077
vet_type	1.320	1	1.149
multiplepartner	1.169	1	1.081
visit_type	1.340	2	1.076

Proportion *Change Talk* Model

QQ plot and residual versus unconditional (on random effects) predictions plot

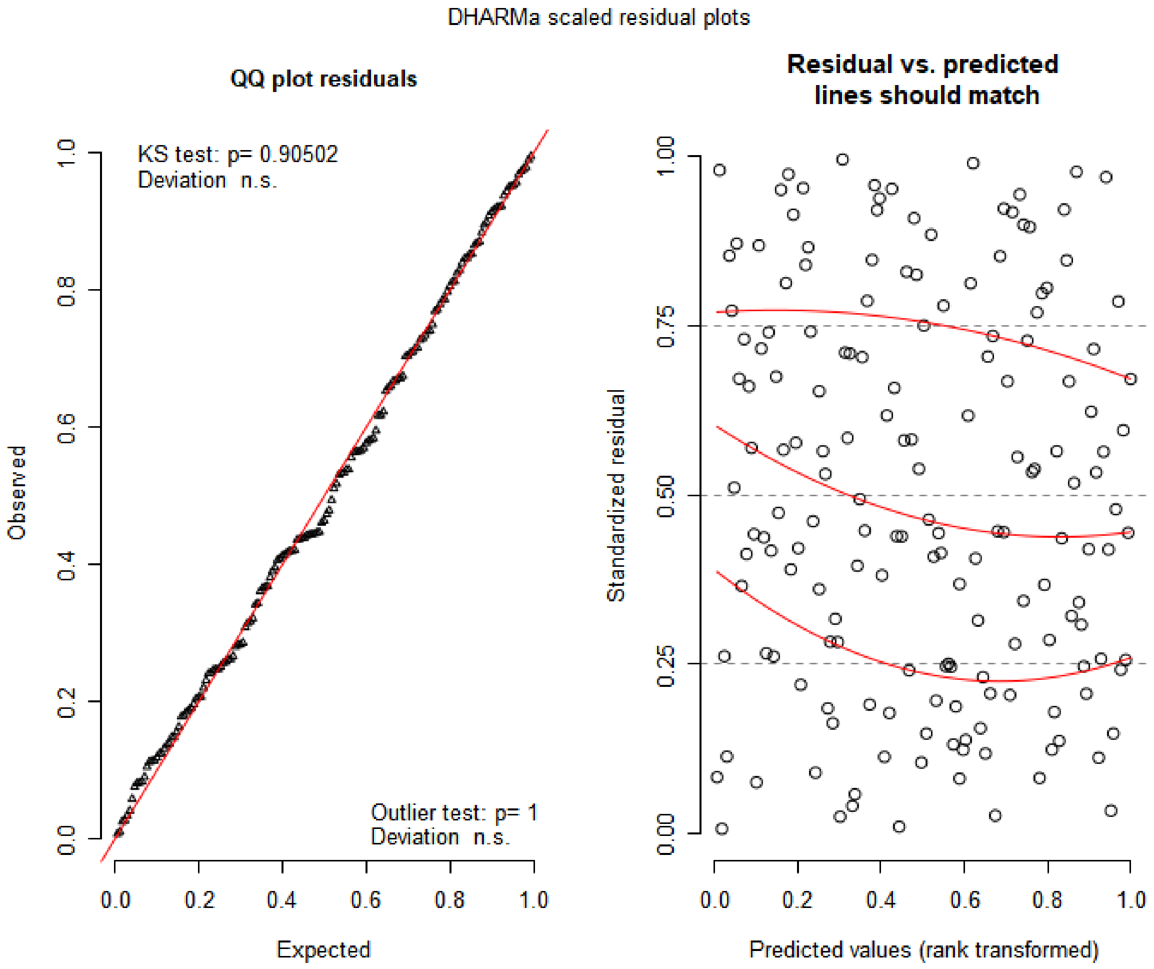

Dispersion test

Estimated dispersion: 0.94, p-value 0.048 (H_0: dispersion $= 1$, H_A: dispersion $\neq 1$)

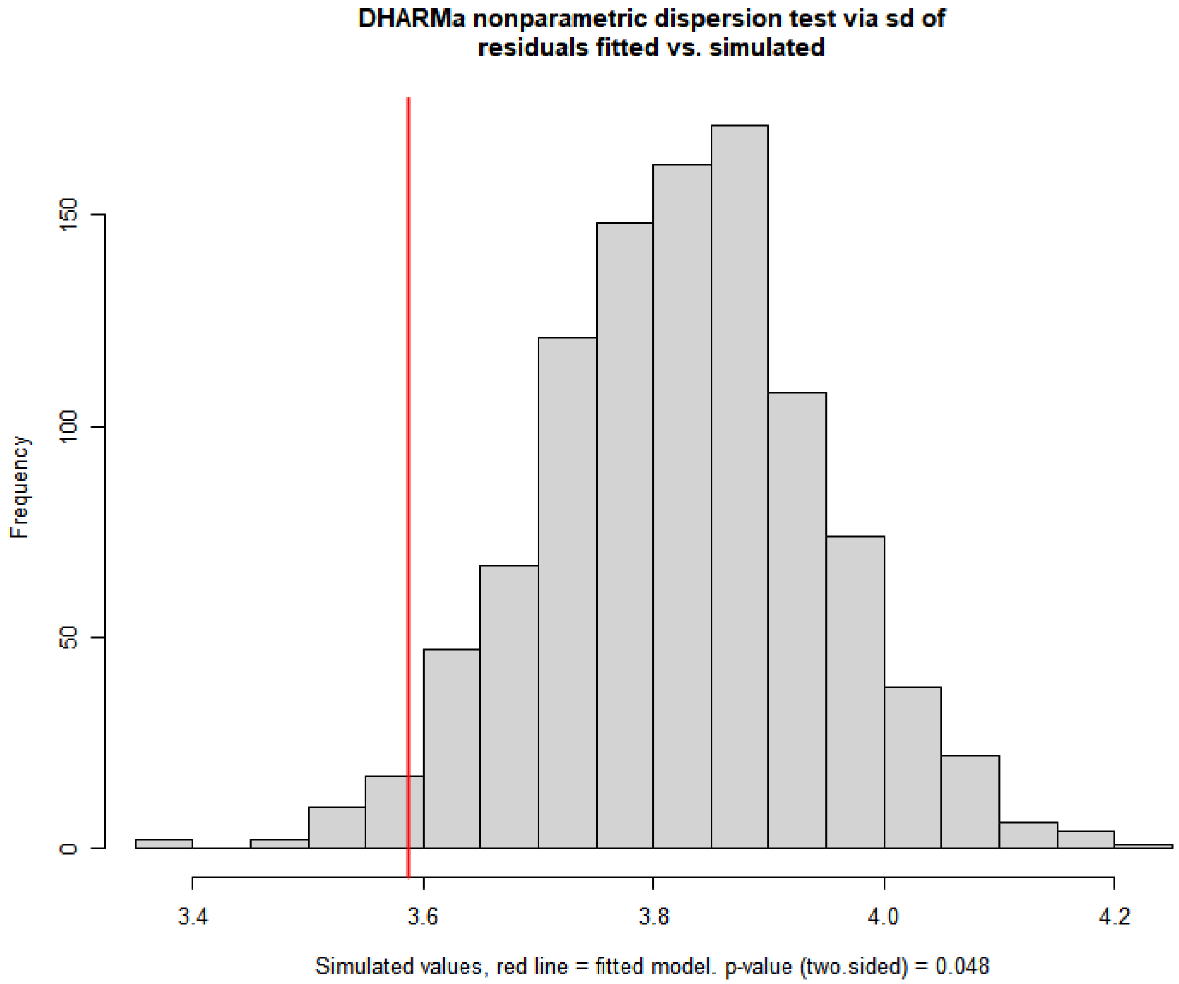

Generalized Variation Inflation Factor (GVIF)

Variable	GVIF	Df	$GVIF^{\frac{1}{2Df}}$
age	1.266	1	1.125
rp_mi_skills	1.889	3	1.112
concordance	1.504	1	1.226
vet_gender	1.262	1	1.123
education	1.173	1	1.083
role	1.450	1	1.204
sufficient_time	1.185	1	1.088
satisfaction	1.093	1	1.045
years_in_vhhm	1.178	1	1.085
vet_type	1.399	1	1.183
multiplepartner	1.249	1	1.118
visit_type	1.399	2	1.088

Supplementary Material S4 – Random effect of client (farm)

The large unexplained client variance may potentially reflect a large between-client variability in inclination to change and potentially also reflects the large variability in preventive measures discussed in the conversations. To study these factors was outside the scope of this study, but would be an interesting topic for further research.

Supplementary Material S5 – Time within veterinarian

As described in Materials and methods, the effect of time within veterinarian was not investigated because of the limited number of observations. Another reason was that we did not anticipate any effect using this material. An effect of time was not anticipated in untrained veterinarians because *Years in VHHM* ($\leq$ 5 years; > 5 years) was not associated with outcome. Associations with time would correspond to an effect of an additional experience in VHHM of less than a year.

In the MI-trained veterinarians, increased communication skills post training would be highly unlikely without any coaching and feedback according to previous studies (*Schwalbe CS, Oh HY and Zweben A 2014. Sustaining motivational interviewing: a meta-analysis. Addiction 109, 1287-1294*).

Sustained skills (i.e. no effect of time) was considered a likely scenario because in the MI training in the present study, workshops were accompanied by sustained coaching and feedback throughout 6-7 months. Furthermore, participants were well aware of expectations to deliver MI consultancies during the study period encouraging preparations before consultations.

Eroding of skills post training would also be a likely scenario. However, according to previous studies skills would most likely have eroded already by 3-6 months post training, with no or only smaller changes later on, i.e. during the period when nearly all consultations were recorded. Such changes would be difficult to detect in our models, because within-veterinarian variation in communication performance is known to be substantial and many veterinarians performed their consultations within a relatively short period of time.

In a follow-up study with a larger number of veterinarians and with each veterinarian performing several consultations at e.g. 3 months intervals post training it would be interesting to investigate the effect of time post training on MI skills and thus possibly also on CLEAR results. One way to do this would be to include a fixed effect of time post training and the interaction Time*MI skills. Another possibility would be to perform repeated measures of MI skills, where each measure would consist of sets of at least 3 recordings from different role-play scenarios coded by MITI.
